# Triple-Negative Breast Cancer: An Update on Neoadjuvant Clinical Trials

**DOI:** 10.1155/2012/385978

**Published:** 2012-01-24

**Authors:** Keith D. Amos, Barbara Adamo, Carey K. Anders

**Affiliations:** ^1^Division of Surgical Oncology, School of Medicine, University of North Carolina at Chapel Hill, Chapel Hill, NC 27599, USA; ^2^Lineberger Comprehensive Cancer Center, School of Medicine, University of North Carolina at Chapel Hill, Chapel Hill, NC 27599, USA; ^3^Department of Human Pathology, Integrated Therapies in Oncology Unit, University of Messina, 98125 Messina, Italy; ^4^Division of Hematology/Oncology, School of Medicine, University of North Carolina at Chapel Hill, Chapel Hill, NC 27599, USA

## Abstract

Triple-negative breast cancer (TNBC) is an aggressive malignancy with a poor prognosis despite the high rates of response to chemotherapy. This scenario highlights the need to develop novel therapies and/or treatment strategies to reduce the mortality associated with TNBC. The neoadjuvant setting provides a model for rapid assessment of treatment efficacy with smaller patient accruals and over shorter periods of time compared to the traditional adjuvant setting. In addition, a clear surrogate endpoint of improved survival, known as pathologic complete response, already exists in this setting. Here, we review current data from completed and ongoing neoadjuvant clinical trials for TNBC.

## 1. Introduction

Triple-negative breast cancer (TNBC) is defined histologically as invasive carcinoma of the breast that lacks staining for estrogen receptor, progesterone receptor, and HER2/neu. Approximately 15–20% of breast cancers illustrate this phenotype [1]. TNBC is associated with high proliferative rates, early recurrence, and poor survival rates. This aggressive disease is insensitive to widely used targeted therapies such as trastuzumab and endocrine therapies, tamoxifen and aromatase inhibitors, which have been effective at reducing breast cancer mortality. Younger women and women of African descent have a high prevalence of TNBC [1]. There are limited and often ineffective therapeutic treatment options for patients with stage IV TNBC.

## 2. The Concept of Neoadjuvant Chemotherapy

The use of neoadjuvant chemotherapy for patients with locally advanced breast cancer has increased significantly over several decades. Neoadjuvant chemotherapy was first used in patients with unresectable or marginally resectable breast cancer [2, 3]. The results from initial studies showed high rates of tumor response and regression. Additional clinical trials were performed with the primary objective of determining whether breast conserving surgery could be offered after neoadjuvant chemotherapy to patients who would have traditionally required mastectomy.

The National Surgical Adjuvant Breast and Bowel Project (NSABP) B-18 study randomized 1,523 women with operable breast cancer to receive 4 cycles of adriamycin and cyclophosphamide either in the preoperative or postoperative setting [4]. This study showed that neoadjuvant chemotherapy improved breast conservation rates (67.8% versus 59.8%). Although there was no difference in overall survival (OS) between neoadjuvant and adjuvant therapy groups, patients treated in the neoadjuvant setting whose tumors obtained a pathologic complete response (pCR) at surgery (defined as no histologic evidence of invasive tumor cells in the breast) showed improved disease-free survival (DFS) and OS rates compared to those with residual disease. The association of pCR with survival outcomes has also been observed by other neoadjuvant studies [5, 6]. Thus, pCR is now considered to be an important endpoint in clinical trials assessing the efficacy of neoadjuvant chemotherapy.

 Just as breast cancer has been classified into subtypes with distinct gene expression and associated clinical outcomes [7, 8], response to neoadjuvant chemotherapy by subtype is also unique. For example, the pCR rate for patients with hormone receptor (HR)-positive tumors was 8% after anthracycline-based or anthracycline/taxane-based chemotherapy [9]. In contrast, the pCR rate for TNBC patients undergoing similar therapies was found to be 25% [9], despite poorer overall outcome when compared to those with HR-positive disease. This phenomenon, termed the “triple negative paradox,” is supported by data from several notable clinical studies; however, the reason for this phenomenon is largely unknown [5, 6, 10].

 Recognizing the clinical heterogeneity of breast cancer, a group of investigators sought to determine if different molecular subclasses of breast cancer responded differently to anthracycline- and paclitaxel-containing preoperative chemotherapy [10]. To answer this question, fine needle aspirations of breast cancer were obtained from 82 patients prior to initiation of neoadjuvant paclitaxel followed by 5-fluorouracil, doxorubicin, and cyclophosphamide chemotherapy. Gene expression profiling was performed and each breast cancer was assigned a unique molecular class—luminal (*n* = 30), basal-like (*n* = 22), and HER2-positive (HER2+; *n* = 20) breast cancers. The rates of pCR, defined as no residual invasive cancer in the breast and axillary lymph nodes, differed significantly among these three molecular classes of breast cancer. Basal-like breast cancers, of which greater than 85% were either estrogen receptor and/or HER2 negative, were associated with high rates of pCR 45% (95% confidence interval (CI) 24–68). Similarly, the HER2+ subgroup was associated with high rates of pCR (45%, CI 23–68), whereas those with luminal tumors illustrated much lower pCR rates (6%, CI 1–21). Genes associated with pCR were examined between the basal-like and HER2+ subtypes, and there was no overlap in these gene sets. This data indicates that genes associated with chemotherapy sensitivity likely differ between these two molecular subgroups of breast cancer.

 Not only has response to preoperative chemotherapy been shown to differ by breast cancer subtype, but also prognosis, particularly as it relates to residual disease following neoadjuvant therapies. Carey et al. sought to examine the relationship between neoadjuvant response and long-term end points, including distant DFS (DDFS) and OS [6]. In this landmark study, 107 patients with stage II-III breast cancer were treated with 4 cycles of neoadjuvant doxorubicin/cyclophosphamide chemotherapy (75% also received preoperative taxanes) between the years 1998 and 2003. Breast cancer subtypes were defined as follows using immunohistochemistry-surrogate markers: 34% for luminal A (HER2−/HR-positive), 24% for luminal B (HER2+/HR-positive), 10% for HER2+ (HER2+/HR negative), and 32% for basal-like (HER2−/HR negative). Similar to the Rouzier et al. study, pCR was higher among patients with basal-like and HER2+ breast cancer (27% and 36%, resp.) and only 7% in luminal breast cancers (*P* < 0.05 in both comparisons). Although pCR was higher among those with HER2+ and basal-like breast cancer, patients of either subtype experienced inferior DDFS and OS compared to luminal breast cancer patients. Overall, only 2 of 17 patients across subtypes with pCR relapsed. Thus, the overall worse outcome observed within basal-like and HER2+ subtypes was due to higher relapse rates among those with residual disease.

 A subsequent analysis conducted by Liedtke et al. performed a similar analysis that evaluated 1,118 patients who received neoadjuvant anthracycline and/or taxane-based chemotherapy at MD Anderson Cancer Center between the years 1985–2004 [5]. In this cohort of patients, 255 patients (23%) were classified as having TNBC, while 863 patients (77%) had non-TNBC. Consistent with prior reports, increased pCR rates were observed for patients with TNBC compared with non-TNBC (22% versus 11%; odds ratio [OR] = 1.53, *P* = 0.034). Despite this difference in pCR, a significant decrease in 3-year RFS and OS was observed for patients with TNBC compared with non-TNBC (63% versus 76%, *P* = 0.0001 and 74% versus 89%, *P* = 0.0001 resp.). Moreover, if a pCR was achieved, patients with TNBC and non-TNBC had similar survival (HR = 1.7, *P* = 0.24). Conversely, patients with residual disease experienced worse OS if they had TNBC compared with non-TNBC (HR = 1.5; *P* < 0.0001). This data supports the continued efforts to identify novel neoadjuvant approaches that will enhance pCR rates among women with TNBC (and non-TNBC). In parallel, there is a need to develop therapeutic strategies for TNBC with residual disease following neoadjuvant therapy.

## 3. Ongoing and Completed Neoadjuvant Therapeutic Strategies for TNBC

As per the most recent National Cancer Comprehensive Network (NCCN) guidelines for the treatment of invasive breast cancer, women with stage IIA–IIIA breast cancer who, with the exception of tumor size, are otherwise candidates for breast-conserving therapy, may be considered for preoperative chemotherapy with a number of anthracycline and/or taxane-based regimens (http://www.nccn.com/). While these chemotherapy regimens remain the mainstay to treat operable TNBC [11], salient efforts are being made to improve outcomes for women diagnosed with this aggressive disease. Some of these strategies include the addition of chemotherapeutic agents to the anthracycline/taxane backbone, as well as the incorporation of biologic and targeted agents to standard regimens. Many of the completed and ongoing clinical trials testing novel neoadjuvant treatment strategies for TNBC will be reviewed here (see Table 1).

## 4. Chemotherapy

Building on experiences in the metastatic setting where select combination chemotherapies have led to improved breast cancer outcomes compared to single agent regimens [20, 21], several neoadjuvant studies have sought to determine the additive benefit of incorporating novel chemotherapeutics with standard anthracycline and/or taxanes. These additional chemotherapeutics have included antimetabolites, platinum agents, and novel microtubule stabilizing agents.

### 4.1. Antimetabolites

The recently reported National Surgical Adjuvant Breast and Bowel Project (NSABP) B-40 protocol asked two fundamental questions: (1) was the addition of the antimetabolite either capecitabine (X) or gemcitabine (G) to docetaxel (T) followed by AC, and/or (2) does the addition of bevacizumab to docetaxel/anthracycline-based regimens increase pCR rates for women with HER2-negative breast cancer [14]. While this study was not restricted with women with TNBC, 41% of the 1,206 patients had HER2-negative/HR-negative breast tumors (thus, triple negative). Complete clinical response as assessed by physical exam was not significantly different by treatment arm (*P* > 0.4). Similarly, no statistically significant difference was observed for pCR in both breast and lymph nodes across all treatment arms: T → AC 26%; TX → AC 23.3%; TG → AC 27.3% (*P* > 0.4; Table 1). Toxicity was reported for 1,191 patients, including all grade 3 and 4 adverse events and was numerically higher for the TX → AC (55% Grade 3 and 14% Grade 4) and TG → AC (61% Grade 3 and 12% Grade 4) arms compared to the T → AC arm (48% Grade 3 and 7% Grade 4).

 A second study sought to determine the additional benefit of preoperative capecitabine to docetaxel—either sequentially or in combination—to treat women with HER2-negative breast cancer [16]. In this study, 51 women were treated with either 4 cycles of docetaxel followed by 4 cycles of capecitabine (Arm A, *n* = 25) or 8 cycles of concurrent docetaxel/capecitabine. Median tumor size was 6.1 cm, 68% of patients were clinically lymph node positive, and 41.2% had TNBC. Overall, treatment was well-tolerated with expected grade 3 and 4 toxicities (15.7% neutropenia, 5.9% neuropathy, and 3.9% neuropathy). For the entire study cohort, pCR rates were 8% and 11.5% for Arm A and B, respectively. Among those with TNBC, pCR rate in both arms combined was 19%.

 While the results of these two studies illustrate modest, at best, activity for the addition of antimetabolites to anthracycline/taxane and/or taxane-based therapy, results as they pertain to TNBC should be interpreted with caution as only 40% of study populations were classified as triple negative. In addition, and given the higher toxicity profile associated with doublet chemotherapy, biomarker strategies to both enrich for responders and minimize toxicities associated with antimetabolites should be considered and incorporated into future neoadjuvant studies examining combination strategies.

### 4.2. Platinum Therapy

Given the inherent genomic instability of TNBC/basal-like with and without *BRCA* germline mutations and respectable sensitivity to platinums in the metastatic setting [22–24], several neoadjuvant studies have evaluated these agents as monotherapy or in different combination strategies. In Silver et al., 28 women with Stage II or III TNBC (of which 2 harbored a germline *BRCA1* mutation) were treated with 4 cycles of cisplatin monotherapy 75 mg/m^2^ every 21 days. The pCR rate was 21% (6/28), and the partial and complete clinical response was 64% (18/28). Several variables were associated with response: young age, low *BRCA1* mRNA expression,* BRCA1* promoter methylation, p53 nonsense or frameshift mutations, and a gene expression signature of E2F3 activation. In a subsequent study of two Polish series of women with *BRCA1*-mutated breast cancer largely triple-negative treated with cisplatin monotherapy (75 mg/m^2^ every 21 days), the pCR rates were as high as 80–90% [13, 25]. Further studies are needed to determine if *BRCA1* mutations are predictive of cisplatin benefit in TNBC.

 The recently reported GEICAM 2006-03-A study sought to determine the additional benefit of carboplatin to conventional neoadjuvant chemotherapy in women with TNBC/basal-like breast cancer patients (defined as ER−/PR−/HER2− and cytokeratin 5/6+ and/or epithelial growth factor receptor [EGFR]+) [15]. In this Phase II multicenter study, 94 patients with ≥2 cm tumors were randomized to receive epirubicin/cyclophosphamide for 4 cycles followed by either docetaxel with or without carboplatin for 4 cycles. pCR in both the breast and axilla was reported to be 30% in both arms; Grade 3/4 toxicities between arms were similar (54% and 53%).

Ongoing studies will continue to help us define the role, timing, and optimal patient population of platinums in the preoperative treatment of TNBC. As an example, the Cancer and Leukemia Group B (CALGB) 40603 clinical trial is actively enrolling patients to standard anthracycline/taxane-based neoadjuvant therapy without carboplatin (NCT00861705). Pretreatment breast core biopsies are required at study entry. Both the clinical outcomes and correlative endpoints of this study will help guide future use of platinum agents in this setting.

### 4.3. Microtubule Stabilizing Agents

Ixabepilone, a novel semisynthetic antineoplastic agent derived from natural epothilones and their analogs, promotes tumor cell death by stabilizing microtubules and inducing cell cycle arrest and subsequent apoptosis. A large, randomized, Phase III study illustrated improvement in PFS by the addition of ixabepilone to capecitabine to treat women with metastatic breast cancer, including those with TNBC [26]. This has led investigators to evaluate the benefit of ixabepilone in the neoadjuvant treatment of invasive breast cancer not amenable to breast conservation surgery [19]. In this study, 161 women with inoperable breast cancer (of which 42 [26%] were triple negative) were treated with 4 or fewer cycles of single agent ixabepilone. pCR rates in the breast were 18% for the entire study population; 22% in ER negative/HER2 negative; 46.1% in ER negative/HER2+; 10.6% in ER positive/HER2-negative; 20% in ER positive/HER2+. Gene expression studies from pretreatment core breast biopsies confirmed the inverse relationship between ER expression and ixabepilone sensitivity. An ongoing clinical trial evaluating differential responses to neoadjuvant paclitaxel versus ixabepilone following AC chemotherapy in the preoperative setting of early stage breast cancer is eagerly awaited (NCT00455533).

## 5. Antiangiogenic Agents

It is well established in both the laboratory and clinical settings that angiogenesis is a key mediator of breast cancer progression [27]. Multiple studies have evaluated the benefit of targeting vascular endothelial growth factor receptor (VEGF) with the humanized monoclonal antibody, bevacizumab (Avastin, Genentech/Roche). Although results were more impressive in the E2100 study as compared to others, the addition of bevacizumab has consistently led to improvements in response rates, while PFS benefit has been more modest [28–30]. However, as some benefit has been seen in the TNBC subset and given the relative paucity of “targets” in TNBC, several investigators have sought to determine the benefit of targeting VEGF with bevacizumab in the neoadjuvant setting (see Table 2).

The GeparQuinto study was designed to determine the benefit to adding bevacizumab to anthracycline/taxane-based preoperative chemotherapy among 1,948 women with HER2-negative breast cancer [32]. Patients were randomized to receive 4 cycles of epirubicin/cyclophosphamide (EC) followed by 4 cycles of docetaxel (D) with or without bevacizumab. Approximately 35% of patients in both arms had TNBC. For the entire study cohort, there was no statistical significant difference in pCR (defined as no invasive/noninvasive residual in breast and nodes) between groups (15% EC → D and 17.5 EC → D plus bevacizumab). In a predefined stratification by subtype, patients with TNBC had a significantly higher likelihood of pCR by the addition of bevacizumab compared to the other subtypes (OR = 1.42). In a subsequent analysis in TNBC patients only (*n* = 684) reported at ASCO 2011 annual meeting, pCR rates in both breast and lymph nodes were higher for patients who received EC → T plus bevacizumab compared to EC → D alone (36.4% versus 28%, *P* = 0.021) [31]. A large biomarker program is ongoing to try to identify subgroups within TNBC who achieve greater benefit from bevacizumab. 

 In addition to evaluating the benefit of adding antimetabolites to standard anthracycline/taxane-based chemotherapy, the recently reported NSABP B-40 study also sought to determine if the addition of bevacizumab would enhance pCR rates for >1,200 women with HER2-negative breast cancer [14]. In this study, patients were treated with AC followed by docetaxel with or without bevacizumab. Complete clinical responses were higher among women who received bevacizumab (64.3 versus 55.8%, *P* = 0.006). This effect was more dramatic in those with HR-positive breast cancer (64.5% versus 53.7% with and without bevacizumab, resp., *P* = 0.007) compared to those with TNBC (63.9% versus 59.1% with and without bevacizumab, resp., *P* = 0.371). Similar to clinical response, pCR was higher for patients who received bevacizumab compared to those who did not (34.5 versus 28.4%; OR = 1.33, *P* = 0.027), and the positive effect was more prominent in patients with HR-positive tumors (OR = 1.7, *P* = 0.008) as compared to those with TNBC (OR = 1.17, *P* = 0.44). Given the apparent differences in response rates between the GeparQuinto and B40 studies within TNBC, the results of the ongoing CALGB study 40603 (NCT00861705) evaluating both the addition to platinum and bevacizumab to standard anthracycline/taxane chemotherapy are eagerly awaited. 

## 6. Novel Targeted Strategies: Small Molecule Inhibitors

In addition to advances in combination chemotherapeutics and antiangiogenic agents, substantial effort is being made to optimize preoperative response rates through the use of novel agents targeting important oncogenic signaling pathways in breast cancer. These strategies include the inhibition of mammalian target of rapamycin (mTOR), histone deacetylase (HDAC), and poly-ADP-ribose polymerase (PARP). 

 Given that activation of the PI3K/mTOR pathway activation occurs frequently in TNBC, investigators sought to determine the benefit of adding RAD001 (Novartis), an mTOR inhibitor, to neoadjuvant anthracycline/taxane chemotherapy [33]. Fifty patients with TNBC were randomized to receive paclitaxel weekly for 12 weeks with or without weekly RAD001 for 12 weeks, both followed by 5FU/epirubicin/cyclophosphamide (FEC) every 3 weeks for 4 cycles. Although pCR rates did not differ by treatment arm (30.4% versus 25.9%, *P* = 0.761), investigators collected breast tumor biopsies to evaluate molecular changes in the PI3K pathway at baseline, 48 hours, 12 weeks after-therapy and at surgery. Ongoing correlative science studies are likely to help refine the selection of patients most likely to respond to these targeted agents. 

 Epigenetic mechanisms are another potential target for TNBC. For example, studies have shown that the loss of ER-*α* by gene methylation might be occurring in ER-negative breast tumors, and that demethylation could restore the expression of ER and sensitize the tumor cells to hormonal therapies [34]. In addition, preclinical and early phase clinical studies have illustrated efficacy for targeting endocrine-resistant breast cancers with HDAC inhibitors [34, 35]. Building on these results, an ongoing study (NCT00262834) is evaluating change in tumor morphology, tissue and blood (peripheral blood mononuclear cells) histone acetylation, and safety of short term exposure to the HDAC inhibitor, vorinostat (Merck), for newly diagnosed breast cancers. These results will undoubtedly inform future trials evaluating HDAC inhibitors in the neoadjuvant treatment of breast cancer. 

 Finally, the I-SPY 2 trial (investigation of serial studies to predict your therapeutic response with imaging and molecular analysis 2) is a multicenter, neoadjuvant study projected to enroll over 800 women with breast cancer of all phenotypes (NCT01042379). This trial is integrating novel imaging and biomarker analysis to improve response prediction to a variety of novel targeted agents in combination with standard chemotherapeutics. Pertinent to TNBC, a subset known to share clinicopathologic features with *BRCA*-deficient breast cancers [36] will be treated with the PARP inhibitor, ABT-888.

## 7. Conclusions 

Although TNBC has an overall poor prognosis, TNBC patients undergoing neoadjuvant chemotherapy have improved breast conservation rates and high response rates. In this setting, pCR is an appropriate endpoint for predicting improved longer-term outcome. However, this endpoint is only achieved by current treatment strategies in 20–40% of the cases. Thus, we recommend that patients presenting with operable TNBC be encouraged to participate in neoadjuvant clinical trials since there are a number of novel targeted agents that are currently being evaluated. 

**Figure 1:**
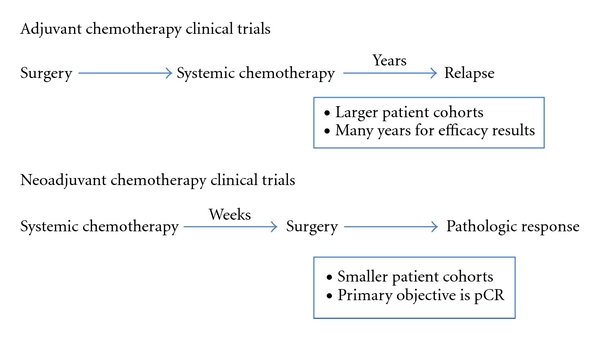
Clinical trial design schematic.

Treatment in the neoadjuvant setting provides an ideal model for evaluating the efficacy of new targeted therapies for TNBC. Such an approach allows for smaller patient accrual, shorter timeframes to obtain results and routine tissue collection for correlative studies compared to traditional adjuvant trials (see Figure 1). Neoadjuvant trials allow for more rapid evaluation of novel therapies for TNBC. In addition, primary tumor core biopsies can be obtained before initiation of systemic therapy and during therapy for correlative studies to assess the status of particular biomarkers and test if the presumed targets are being inhibited by these novel therapies. For example, proliferation-related biomarker Ki-67 has been shown to be a useful surrogate for response during or after neoadjuvant endocrine therapy [37]. 

 In closing, there are numerous ongoing clinical neoadjuvant trials aimed at improving outcome for patients with TNBC. Moreover, the use of neoadjuvant chemotherapy as the primary model for clinical research for TNBC will advance our understanding of molecular response to novel agents and our ability to efficiently assess the efficacy of promising therapies with the ultimate goal of improving patient survival.

## Figures and Tables

**Table 1:** Summary of completed neoadjuvant chemotherapy trials.*

Clinical trials	Design	Drugs	Population	pCR rate
Silver et al. [12]	Phase II single arm	Cisplatin × 4	TNBC	6/28 (21%)

Byrski et al. [13]	Retrosp.	All; CMF; AD; AC/FAC; cisplatin	*BRCA1* mut.	All: 24/102 (24%) CMF: 1/14 (7%) AD: 2/25 (8%) AC/FAC: 11/51 (22%) Cisplatin: 10/12 (83%)

Bear et al. [14]	Phase III random.	Arm 1A: D × 4 → AC × 4 Arm 1B: D + X × 4 → AC × 4 Arm 1C: D + G × 4 cycles → Ac × 4	HER2−	Arm 1A: 102/393 (26%) Arm 1B: 91/390 (23%) Arm 1C: 106/388 (27%)

Alba et al. [15]	Phase II random.	Arm A: EC × 4 cycles → D × 4 Arm B: EC × 4 cycles → D + Carbo × 4	Basal-like	Arm A: 14/46 (30%) Arm B: 14/47 (30%)

Zelnak et al. [16]	Phase II random.	Arm A: D × 4 cycles → X × 4; Arm B: D + X × 8 cycles.	HER2−	Arm A: 2/25 (8%) Arm B: 3/26 (12%) Arm A/B (TNBC): 4/21 (19%)

Von Minckwitz et al. [17] Huober et al. [18]	Phase III random.	Arm 1 (responder): TAC × 4 Arm 2 (responder): TAC × 6 Arm 3 (nonresponder): TAC × 4 Arm 4 (nonresponder): VX × 4	Any breast cancer	Arm 1–4 TNBC: 77/198 (39%) Non-TNBC: 22/147 (15%)

Baselga et al. [19]	Phase II single arm	Ixabepilone × 4	Any breast cancer	TNBC: 11/42 (26%) Non-TNBC: 18/119 (15%)

*TNBC: triple-negative breast cancer; pCR: pathological complete response; M: methotrexate; F: 5-fluorouracil; Retrosp.: retrospective study; T: paclitaxel; Carbo: carboplatin; D: docetaxel; C: cyclophosphamide; A: doxorubicin; E: epirubicin; X: capecitabine; G: gemcitabine; V: vinorelbine.

**Table 2:** Summary of neoadjuvant bevacizumab-based chemotherapy trials.*

Clinical trials	Design	Drugs	Population	Status	pCR rate
Gerber et al. [31] (GeparQuinto)	Phase III	Arm 1: EC × 4 → D × 4 Arm 2: EC+ Bev × 4 → D + Bev × 4	TNBC	Completed	Arm 1: 96/342 (28%) Arm 2: 119/327 (36.4%)

Bear et al. [14] (NSABP B-40)	Phase III random	Arm 1A-C: Anthracycline-taxane-based chemotherapy Arm 2A-C: Anthracycline-taxane-based chemotherapy + Bev	HER2−	Completed	All Arms Bev: 203/588 (35%) All Arms/no Bev: 168/592 (28%) TNBC Bev: 121/236 (51%) TNBC/no Bev: 115/243 (47%) HR+ Bev: 82/352 (23%) HR+/no Bev: 53/349 (15%)

CALGB-40603	Phase II random	Arm 1: T → AC Arm 2: T + Bev → AC + Bev Arm 3: T + Carbo → AC Arm 4: T + Carbo + B → AC + Bev	TNBC	Ongoing	—

*TNBC: triple-negative breast cancer; pCR: pathological complete response; Bev: bevacizumab; T: paclitaxel; Carbo: carboplatin; D: docetaxel; C: cyclophosphamide; A: doxorubicin; E: epirubicin.
